# Learning Compact Identity Representations for Weakly Textured Hanwoo Cattle Re-Identification

**DOI:** 10.3390/ani16152320

**Published:** 2026-07-28

**Authors:** Jiaqi Liu, Alvaro Fuentes, Shujie Han, Sook Yoon, Yongchae Jeong, Dong Sun Park

**Affiliations:** 1 Department of Electronics Engineering, Jeonbuk National University, Jeonju 54896, Republic of Korea; 2 Core Research Institute of Intelligent Robots, Jeonbuk National University, Jeonju 54896, Republic of Korea; 3 Department of Computer Engineering, Mokpo National University, Muan 58554, Republic of Korea

**Keywords:** animal re-identification, Hanwoo cattle, weakly textured objects, identity compactness, conditional diffusion, viewpoint-aware feature centralization

## Abstract

Cross-view re-identification of Hanwoo cattle remains challenging: weak texture provides only limited identity cues, and large pose and viewpoint changes easily distort these cues, causing severe feature dispersion. As a result, samples from the same individual may be scattered in the feature space, whereas visually similar individuals can form ambiguous local neighborhoods. To address this problem, we propose a framework that explicitly improves identity compactness for weakly textured cattle ReID. Specifically, Pose- and Text-Conditioned Inpainting Diffusion (PTID) is trained to generate pose-diverse yet identity-consistent samples and, during inference, aggregates their features with the original input feature to approximate a compact identity center and reduce pose-induced feature dispersion. Dual-Adaptive Viewpoint-Aware Feature Centralization (DVFC) further refines retrieval features by adaptively exploiting viewpoint consistency and neighborhood reliability, thereby suppressing unreliable neighbor mixing under large cross-view variation. We also construct a Hanwoo ReID dataset containing 37 identities and 12,480 images collected from three farm environments with substantial variations in pose, illumination, occlusion, and background conditions. Extensive experiments on multiple strong baselines under both closed-set and open-set settings demonstrate consistent improvements, with gains of up to 40.8 points in mAP and 26.1 points in Rank-1 accuracy. These findings demonstrate that explicitly improving identity compactness through identity-consistent generation and viewpoint-aware feature centralization is a promising solution for the robust re-identification of weakly textured cattle.

## Simple Summary

The reliable identification of individual cattle is important for precision livestock farming because it supports the long-term monitoring of animal behavior, health, and welfare. However, automatically identifying Hanwoo cattle is challenging because different individuals often have very similar body appearance, and their visual features can change greatly with pose and camera viewpoint. To address this problem, this study proposes a visual re-identification framework that combines pose-guided image generation with viewpoint-aware feature refinement. Specifically, the method learns to generate pose-diverse, identity-consistent images and, during inference, aggregates their features with the original image features to obtain a more compact identity representation, which is further refined using viewpoint information. Experimental results show that the method improves cattle re-identification performance across different datasets and evaluation settings. This work provides a useful technical reference for developing non-invasive, camera-based cattle identification systems in real farm environments.

## 1. Introduction

Animal re-identification (ReID) becomes particularly difficult when identity cues are weak and unstable. This is exactly the case in Hanwoo cattle ReID, where different individuals often share highly similar appearances and pose changes, viewpoint variation, and cluttered farm backgrounds can severely distort the limited discriminative cues that remain [1]. Under such conditions, samples from the same individual are easily dispersed in the feature space, and visually similar individuals can form ambiguous local neighborhoods, making reliable cross-view retrieval highly challenging.

**Figure 1:**
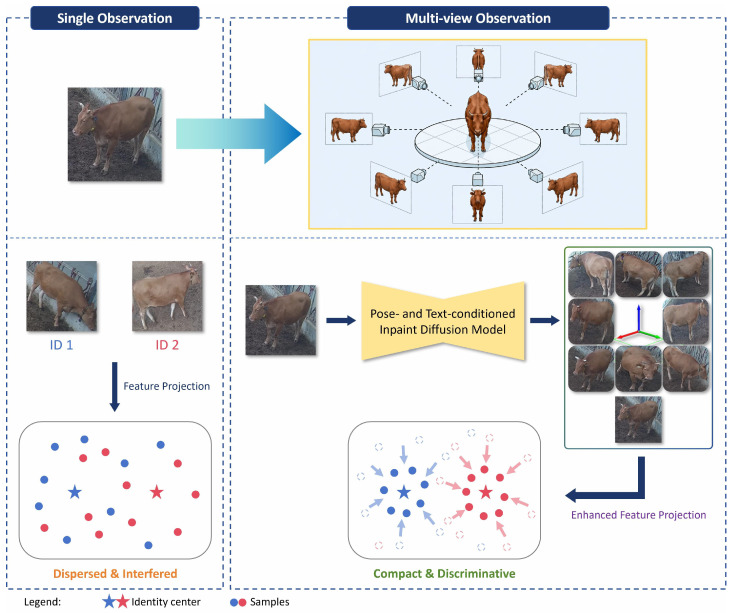
Motivation for the proposed framework in cattle ReID. A single observation may yield an identity feature that deviates from the underlying identity center under pose and viewpoint variations. During inference, aggregating the original feature with features extracted from pose-diverse images generated by PTID produces a more compact and discriminative representation, pulling it toward a more consistent identity center.

Existing ReID methods have shown encouraging performance by learning global appearance representations from full-body images [2,3]. In general, commonly used objectives such as cross-entropy, triplet loss, and center loss encourage intra-class compactness while enlarging inter-class separation [4,5,6]. Nevertheless, directly achieving this goal is non-trivial for cattle ReID under weak texture and large viewpoint variation. In particular, although pose-guided generation has shown promise in pedestrian ReID [7], directly transferring such strategies to cattle is difficult because fine-grained identity cues, such as horn shape, collars, ear tags, and subtle local coat patterns, are often hard to preserve under large pose transformations. As illustrated in Figure 1, a single observation may yield dispersed and interfered identity features in the embedding space, as a lone viewpoint often fails to capture the full appearance of an individual subject, thereby undermining reliable identity discrimination in ReID tasks. This limitation motivates the consideration of multi-view observations, where images captured from diverse angles provide complementary and view-consistent visual cues. By aggregating such multi-view information, the model is able to construct a more compact, coherent, and discriminative feature representation in the embedding space—a property that is particularly critical for robust ReID matching across varying viewpoints and appearances, as commonly encountered in real-world cattle ReID scenarios.

Motivated by this observation, we develop a robust ReID framework that improves identity compactness from two complementary perspectives. First, Pose- and Text-Conditioned Inpainting Diffusion (PTID) reduces pose-induced intra-identity variation. During training, PTID learns to generate identity-preserving images under diverse target poses using a reference image, a pose condition, and a structured text prompt. During inference, the generated pose-diverse samples are aggregated with the original input at the feature level to produce a more compact identity representation. Second, Dual-Adaptive Viewpoint-Aware Feature Centralization (DVFC) further refines retrieval features by estimating instance viewpoints, assessing neighborhood reliability, and adaptively combining local viewpoint-aware support with global contextual support. This process suppresses unreliable feature mixing across inconsistent viewpoints and improves inter-identity separability. In this way, PTID reduces intra-identity variation through pose-guided generation and feature aggregation, whereas DVFC enhances inter-identity discrimination through viewpoint-aware neighborhood refinement.

To support systematic evaluation in realistic settings, we also construct a Hanwoo ReID dataset collected from three farm environments: Imsil, Namwon, and Chilbo. The dataset contains 37 identities and 12,480 images with substantial variations in viewpoint, pose, illumination, and occlusion, enabling both closed-set and open-set evaluation. Extensive experiments on multiple backbones show that the proposed framework consistently improves strong baselines across different sequences and evaluation protocols. These results indicate that combining identity-consistent multi-pose generation and feature aggregation with viewpoint-aware feature centralization is effective for robust ReID of weakly textured cattle in real-world environments.

The main contributions of this work are summarized as follows:

- We propose a Pose- and Text-Conditioned Inpainting Diffusion (PTID) model that combines reference appearance, structural text descriptions, and representative cattle poses to generate pose-diverse, identity-consistent images. During inference, features from the original and generated images are aggregated to improve identity compactness.
- We introduce a Dual-Adaptive Viewpoint-Aware Feature Centralization (DVFC) strategy that estimates cattle viewpoint from hoof geometry and evaluates neighborhood reliability using margin-based distinctiveness, reciprocal consistency, and viewpoint coherence. Based on this reliability, DVFC adaptively balances viewpoint-aware local support and global contextual support to refine retrieval features.
- We construct a multi-farm Hanwoo ReID dataset containing 37 identities and 12,480 images across three environments, supporting systematic evaluation under realistic weak-texture and cross-view conditions.

## 2. Materials and Methods

**Figure 2:**
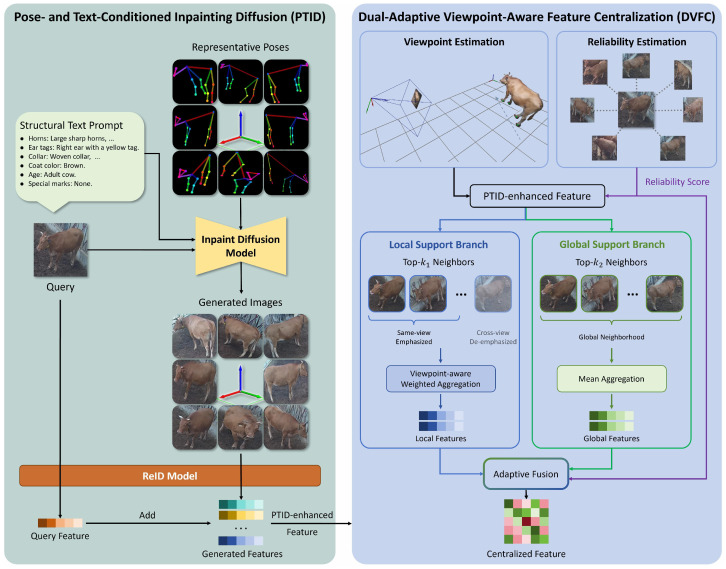
Overview of the proposed framework. PTID generates pose-diverse, identity-consistent images and fuses their features with the original input to enhance identity representation. DVFC further refines the enhanced representation through viewpoint estimation, reliability estimation, and adaptive local–global feature fusion.

To address the challenges of weakly textured cattle ReID under substantial pose and viewpoint variations, we use pose-guided generation to enhance identity representation at inference time through feature aggregation. As illustrated in Figure 2, the proposed framework consists of two sequential components: Pose- and Text-Conditioned Inpainting Diffusion (PTID) and Dual-Adaptive Viewpoint-Aware Feature Centralization (DVFC). Given an input image, PTID uses representative poses and a structural text prompt to synthesize multiple pose-diverse images of the same cattle instance. The features of the generated images are then fused with the input features to obtain an enhanced identity representation. DVFC then further refines the fused features by incorporating viewpoint information, neighborhood reliability, and adaptive local–global support, thereby suppressing unreliable feature aggregation at inference time.

### 2.1. Pose- and Text-Conditioned Inpainting Diffusion

PTID is designed to enrich cross-pose observations while preserving fine-grained identity cues that are easily degraded under large pose variation and partial occlusion. The generation process is guided by two complementary conditions: representative poses, which provide diverse structural targets, and structural text prompts, which preserve fine-grained identity cues.

#### 2.1.1. Representative Poses

To provide diverse structural guidance without introducing excessive redundancy, we construct a compact set of representative poses for each farm. Instead of selecting poses separately for each identity, the representative poses are shared by all cattle instances within the same farm, so that PTID can generate pose-diverse images under a consistent set of target pose conditions.

Following pose estimation, we use the four hoof keypoints of each cattle instance, namely left front (LF), right front (RF), left rear (LR), and right rear (RR), to estimate body orientation. Since these hoof points are assumed to lie on the ground plane, each 2D hoof point is back-projected into the bird’s-eye-view (BEV) plane using the camera geometry. Let $p_{i}=\left(u_{i},v_{i}\right)$ denote the image coordinate of hoof point *i*, and let ${\tilde{p}}_{i}={\left(u_{i},v_{i},1\right)}^{⊤}$ be its homogeneous coordinate. Given the camera intrinsic matrix K and extrinsic parameters (R,t), the corresponding BEV coordinate is computed as

(1) $$q_{i}=\lambda_{i}R^{⊤}K^{-1}{\tilde{p}}_{i}+c,$$

where $c=-R^{⊤}t$ denotes the camera center in world coordinates, and $\lambda_{i}$ is chosen such that $q_{i}$ lies on the ground plane.

The body orientation is then estimated in BEV using the hoof configuration. Specifically, we compute the midpoint of the front hooves and the midpoint of the rear hooves, and use the vector from the rear midpoint to the front midpoint as the body direction. The full orientation range is uniformly divided into eight bins, each covering an angular interval of $45^{\circ }$. One representative pose is manually selected from each bin, resulting in the eight representative poses shown in the figures. In this way, the selected representative poses provide complementary pose conditions for PTID generation while avoiding excessive repetition among similar body orientations.

#### 2.1.2. Multimodal Conditioning and Inpainting Guidance

PTID performs generation with conditioning information that mainly includes representative poses and structural text prompts. Representative poses provide diverse target body configurations, while structural text prompts provide identity-related cues that help preserve fine-grained characteristics during generation. Together, they enable PTID to introduce structural variation while maintaining identity consistency across generated samples.

**Figure 3:**
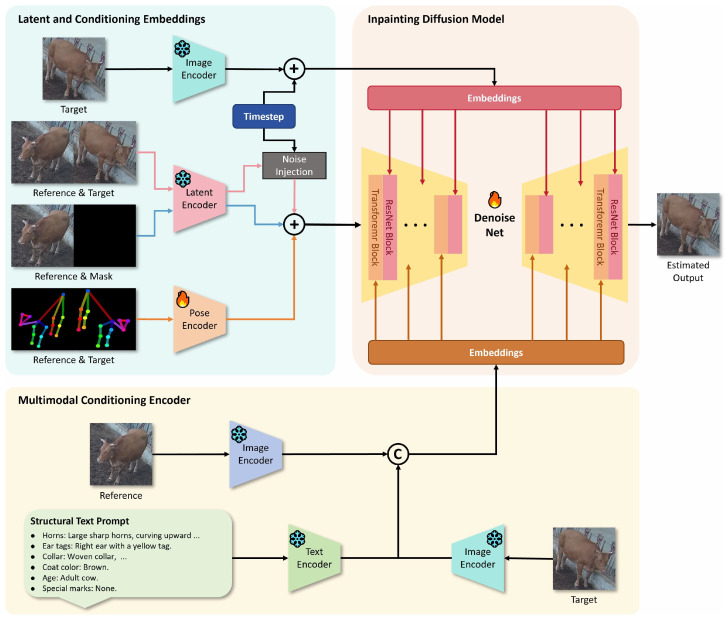
Training architecture of the proposed PTID, consisting of three components: (1) a Latent and Conditioning Embedding module that encodes the target image, masked reference image, and pose into latent and pose-guided signals; (2) a Multimodal Conditioning Encoder that fuses reference images, target images, and structured text into cross-attention embeddings and (3) an Inpainting Diffusion Model that performs identity-consistent inpainting guided by latent and multimodal conditions.

As illustrated in Figure 3, PTID is organized into three modules: a latent and conditioning embedding module, a multimodal conditioning encoder, and an inpainting diffusion model. The first two modules encode the image, pose, and text inputs into conditioning embeddings, which are then injected into the inpainting diffusion model for identity-consistent generation.

During PTID training, given a reference image and a target image, visual features are extracted from both images, while structural text prompts are encoded by a pretrained text encoder. The resulting reference-image, target-image, and structural-text features are concatenated to form a global multimodal condition,

(2) $$f_{\mathrm{mm}}=\mathrm{concat}\left(f_{\mathrm{ref}},\;f_{\mathrm{tgt}},\;f_{\mathrm{txt}}\right),$$

where $f_{\mathrm{ref}}$, $f_{\mathrm{tgt}}$, and $f_{\mathrm{txt}}$ denote the encoded features of the reference image, target image, and structural text prompts, respectively.

For pose conditioning, cattle pose is estimated using RTMPose [8], pretrained on the AP-10K dataset [9]. The resulting pose map is fed into a four-layer convolutional pose encoder to produce the pose conditioning feature $p_{\mathrm{st}}$. In parallel, the masked input is encoded as an image–mask condition $i_{\mathrm{sm}}$, which specifies the target region for inpainting generation. Together, the global multimodal condition $f_{\mathrm{mm}}$, the pose condition $p_{\mathrm{st}}$, and the image–mask condition $i_{\mathrm{sm}}$ guide the inpainting diffusion model to generate pose-diverse yet identity-consistent images.

#### 2.1.3. Diffusion Objective

Given the global multimodal condition $f_{\mathrm{mm}}$, the pose conditioning feature $p_{\mathrm{st}}$, and the image–mask condition $i_{\mathrm{sm}}$, PTID is optimized using the standard inpainting diffusion objective:

(3) $$L_{\mathrm{inpaint}}=E_{x_{0},\epsilon ,f_{\mathrm{mm}},p_{\mathrm{st}},i_{\mathrm{sm}},\tau }\left[{∥\epsilon -\epsilon_{\theta }\left(x_{\tau },f_{\mathrm{mm}},p_{\mathrm{st}},i_{\mathrm{sm}},\tau \right)∥}_{2}^{2}\right],$$

where τ denotes the diffusion timestep.

Once PTID has been trained, no target image is required during inference. Given a reference image, the trained PTID uses its structural text prompt and multiple representative poses to synthesize pose-diverse yet identity-consistent images. Let $z_{i}$ denote the feature of the reference image extracted by the ReID model, and let $z_{i,m}*{m=1}^{M}$ denote the features of the *M* generated images. The PTID-enhanced feature is then obtained by

(4) $$z_{i}^{\mathrm{PTID}}=\mathrm{Normalize}\;\left(z_{i}+\frac{1}{M}\sum_{m=1}^{M}z_{i,m}\right).$$

Through this process, PTID integrates the original observation with pose-diverse generated observations to approximate a more compact identity representation.

### 2.2. Dual-Adaptive Viewpoint-Aware Feature Centralization

**Figure 4:**
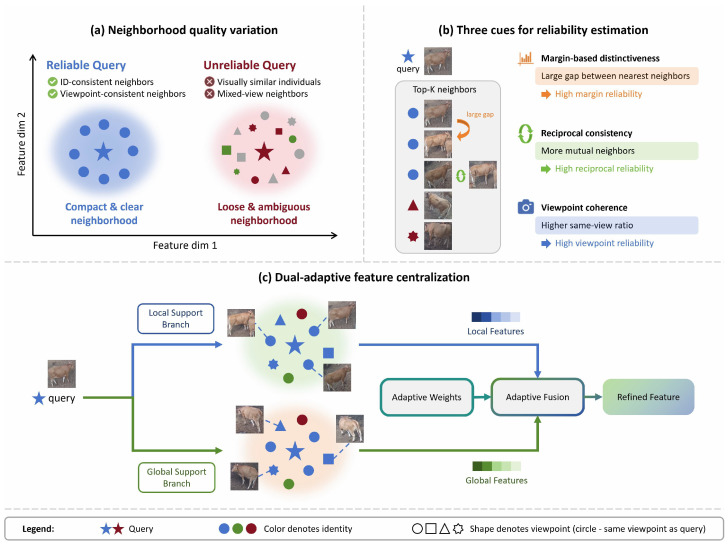
Overview of the proposed Dual-Adaptive Viewpoint-Aware Feature Centralization (DVFC). (**a**) Queries may exhibit different neighborhood qualities in the feature space. (**b**) Reliability is estimated from three complementary cues. (**c**) The query feature is refined by adaptively combining local viewpoint-aware support and global contextual support.

Although PTID improves feature compactness, local neighborhoods may still be unreliable when visually similar cattle are observed from substantially different viewpoints. DVFC addresses this issue by refining the PTID-enhanced features through three consecutive steps: viewpoint estimation, reliability estimation, and adaptive local–global refinement. As illustrated in Figure 4, viewpoint estimation provides geometric cues for feature refinement, reliability estimation assesses the quality of the local neighborhood, and the resulting reliability score is used to adaptively balance local and global support.

#### 2.2.1. Viewpoint Estimation

We first estimate the viewpoint of each instance to determine whether nearby samples are geometrically compatible for local refinement. Specifically, the 2D hoof keypoints are back-projected into 3D space using the camera parameters (K,R,t), and the resulting hoof geometry is used to infer body orientation in BEV. The orientation is then discretized into viewpoint categories. These viewpoint labels are used to favor same-view neighbors and suppress cross-view mismatch in the subsequent refinement stage.

#### 2.2.2. Reliability Estimation

Since the relative contributions of the local and global support branches should depend on the quality of the local neighborhood, we estimate a reliability score $r_{i}$ for each PTID-enhanced feature $z_{i}^{\mathrm{PTID}}$ based on its top-$k_{1}$ nearest neighbors. Let $N_{i}$ denote the indices of these neighbors. The score $r_{i}$ is computed from three complementary cues: margin-based distinctiveness, reciprocal consistency, and viewpoint coherence.

The first cue measures margin-based distinctiveness by evaluating how clearly the nearest neighbor is separated from the remaining neighbors. Let $d_{i,j}$ denote the distance between $z_{i}^{\mathrm{PTID}}$ and its *j*-th nearest neighbor. The corresponding score is defined as

(5) $$r_{i}^{\mathrm{margin}}=\frac{\frac{1}{k_{1}-1}\sum_{j=2}^{k_{1}}d_{i,j}-d_{i,1}}{\frac{1}{k_{1}}\sum_{j=1}^{k_{1}}d_{i,j}+\epsilon }.$$

A larger value of $r_{i}^{\mathrm{margin}}$ indicates that the nearest neighbor is more clearly separated from the remaining neighbors relative to the local neighborhood scale, suggesting a less ambiguous local neighborhood.

The second cue measures reciprocal consistency based on neighborhood agreement. Specifically, $r_{i}^{\mathrm{recip}}$ is computed as the average overlap ratio between $N_{i}$ and the neighborhoods of its top-$k_{1}$ neighbors. A larger value of $r_{i}^{\mathrm{recip}}$ indicates a more stable local neighborhood structure.

The third cue measures viewpoint coherence. Specifically, $r_{i}^{\mathrm{view}}$ is computed as the proportion of neighbors in $N_{i}$ that share the same estimated viewpoint as sample *i*. A larger value of $r_{i}^{\mathrm{view}}$ indicates stronger viewpoint consistency within the local neighborhood.

The final reliability score is defined as

(6) $$r_{i}=\lambda_{1}r_{i}^{\mathrm{margin}}+\lambda_{2}r_{i}^{\mathrm{recip}}+\lambda_{3}r_{i}^{\mathrm{view}},$$

where $\lambda_{1}$, $\lambda_{2}$, and $\lambda_{3}$ are balancing coefficients. Overall, $r_{i}$ summarizes whether the neighborhood of $z_{i}^{\mathrm{PTID}}$ is distinctive, structurally stable, and viewpoint-consistent enough to support strong local refinement.

#### 2.2.3. Adaptive Local–Global Refinement

Based on the estimated viewpoint and reliability score $r_{i}$, DVFC refines each PTID-enhanced feature through two complementary branches: a local support branch and a global support branch. The local support branch aggregates the top-$k_{1}$ nearest neighbors in a viewpoint-aware manner, assigning larger weights to viewpoint-consistent neighbors and smaller weights to viewpoint-inconsistent ones. The global support branch, in contrast, computes the mean feature of the top-$k_{2}$ nearest neighbors and provides a more stable contextual prior.

The contributions of the two branches are then adaptively controlled by the reliability score:

(7) $$\alpha_{i}=\alpha_{\min}+r_{i}\left(\alpha_{\max}-\alpha_{\min}\right),\;\beta_{i}=\beta_{\max}-r_{i}\left(\beta_{\max}-\beta_{\min}\right),$$

where $\alpha_{i}$ and $\beta_{i}$ denote the aggregation weights for the local and global branches, respectively. Accordingly, reliable samples receive stronger local refinement, whereas ambiguous samples rely more on global contextual support.

The final refined feature is obtained by

(8) $${\tilde{z}}_{i}=\mathrm{Normalize}\;\left(z_{i}^{\mathrm{PTID}}+\alpha_{i}z_{i}^{\mathrm{local}}+\beta_{i}z_{i}^{\mathrm{global}}\right).$$

Overall, DVFC refines the PTID-enhanced representation by adaptively balancing viewpoint-aware local evidence and global contextual support according to the reliability of the local neighborhood.

## 3. Results

### 3.1. Dataset and Evaluation Protocols

The dataset used in this study extends that previously reported in Hanwoo ReID [10] by incorporating additional cattle images collected from Chilbo Farm. Structural text prompts and viewpoint-related metadata were also added to support the proposed method. The videos were captured using Hikvision cameras (Hangzhou Hikvision Digital Technology Co., Ltd., Hangzhou, China). During dataset construction, the continuous videos were temporally subsampled according to the motion characteristics of the cattle to reduce redundancy between neighboring frames, and heavily occluded instances were removed when the IoU between the bounding boxes exceeded 0.5. The retained images were then randomly partitioned into the training, query, and gallery sets according to the evaluation protocols described below. The dataset comprises three farm environments, namely Imsil, Namwon, and Chilbo, covering seven daytime sequences with 37 identities and 12,480 images in total. As summarized in Table 1, these environments differ substantially in camera deployment, image resolution, and scene complexity, thereby providing diverse conditions for livestock ReID in real-world settings.

**Figure 5:**
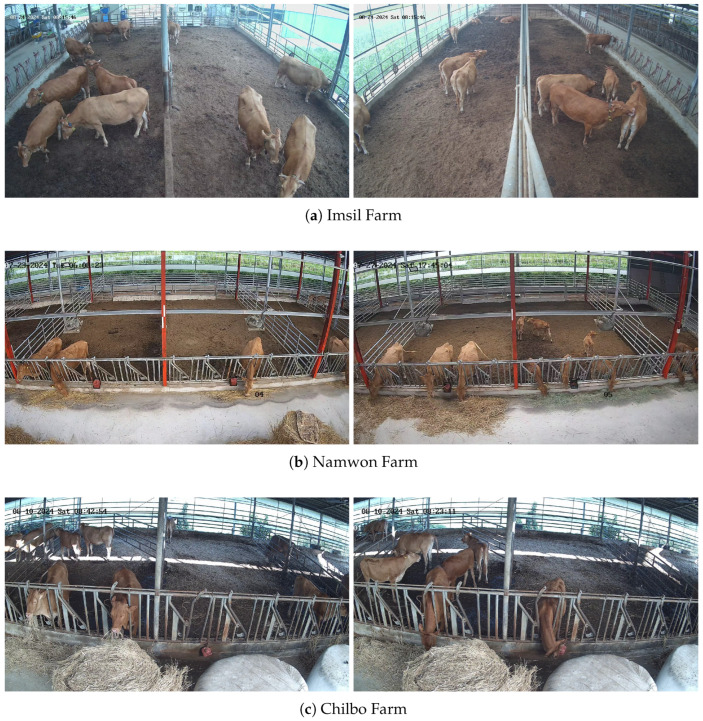
Representative images from the three farm environments in the Hanwoo ReID dataset.

Representative images from the three farm environments are shown in Figure 5. Imsil Farm contains a relatively large number of cattle within the camera view, which increases inter-instance clutter and visual interference among similar animals. In Namwon Farm, the cameras are installed farther from the cattle, making individual instances smaller and less visually clear. In Chilbo Farm, the cameras are located close to the fences, leading to frequent railing occlusions and partial visibility of cattle bodies. These scene-specific factors introduce practical challenges for robust cattle ReID across real farms.

Each cattle instance is assigned a consistent identity label across views. In addition, all structural text prompts were manually annotated using brief descriptions of the appearance and structural characteristics visible in each image, including horn shape, ear tags, collars, coat color, age, and special marks. Experiments are conducted under both closed-set and open-set protocols, with the train/query/gallery splits summarized in Table 2. In the closed-set setting, the training and testing identities overlap. For the Imsil subsets, the training set is constructed by merging the train images from the four Imsil sequences, while query and gallery images are evaluated separately for each sequence. Namwon-0722 is included in the full dataset statistics but is not used for closed-set evaluation.

In this study, the term “open-set” refers to an identity-disjoint protocol in which the training identities are completely disjoint from those in the query and gallery sets, while every query identity is represented in the gallery. For the Imsil open-set subsets, the training set is constructed by merging images of the training identities from the four Imsil sequences, while query and gallery images are taken from the corresponding test sequence using unseen identities. For Namwon-0722*, the nine training identities are taken from Namwon-0727, while the query and gallery identities are from Namwon-0722. Since these two subsets were collected from different areas of the Namwon farm, this setting further reflects a cross-scene ReID scenario. For each evaluated subset, the gallery-to-query ratio is kept close to 5:1 where possible to maintain comparable retrieval difficulty. All experiments are evaluated using standard ReID metrics, including mean Average Precision (mAP) and Cumulative Matching Characteristic (CMC) at Rank-1 and Rank-5.

### 3.2. Implementation Details

We evaluate the proposed PTID and DVFC modules on four ReID models: TransReID [11], RotTrans [12], PFD [13], and HanwooReID [10]. For fair comparison, all models are evaluated under the same data splits and evaluation protocols.

For PTID training, we construct all ordered reference–target image pairs within each identity, such that each image is paired with every other image of the same identity. The inpainting diffusion model is built upon pretrained Stable Diffusion v2.1 [14]. For multimodal conditioning, we use CLIP ViT-H/14 [15] as the text encoder for structural text prompts and DINOv2-Giant [16] as the image encoder for extracting reference-image and target-image features.

RTMPose [8] is used to extract 17 pose keypoints from each cattle image. A hoof keypoint is considered reliably detected when its confidence score is at least 0.30. Among the 12,480 evaluated images, all four hoof keypoints are reliably detected in 11,276 images, corresponding to a four-hoof detection rate of approximately 90.35%. All predicted keypoints are retained for skeleton construction without additional filtering or manual correction.

All experiments were performed on an NVIDIA H100 GPU (NVIDIA Corporation, Santa Clara, CA, USA). A fixed random seed was used to improve reproducibility. The model is trained for 20,000 iterations with a batch size of 64 using the AdamW optimizer, with a learning rate of $2.5\times 10^{-5}$, weight decay of $1\times 10^{-2}$, and a warm-up of 5000 steps. All input images are resized to 256×256. Under this configuration, the peak GPU memory usage is approximately 80 GB.

For DVFC, the neighborhood sizes of the local and global support branches are set to $k_{1}=3$ and $k_{2}=3$, respectively, providing compact neighborhood support while limiting the inclusion of potentially unreliable neighbors. The three reliability cues are combined using fixed weights $\lambda_{1}=0.4$, $\lambda_{2}=0.4$, and $\lambda_{3}=0.2$, with equal weights assigned to the two primary cues and a smaller weight assigned to the auxiliary cue. The adaptive weight ranges are set to $\alpha_{\min}=0.05$, $\alpha_{\max}=0.35$, $\beta_{\min}=0.65$, and $\beta_{\max}=1.00$ across all experiments, thereby controlling the contributions of the local and global branches while retaining the PTID-enhanced feature as the base representation Since $k_{1}$ and $k_{2}$ directly determine the neighborhood scales used for feature refinement, they are treated as the primary hyperparameters and are further analyzed in the ablation study.

### 3.3. Quantitative Results

#### 3.3.1. Closed-Set Evaluation

Under the closed-set setting, we evaluate the proposed modules on six sequences. As shown in Table 3 and Table 4, PTID consistently improves the baseline performance, and DVFC further provides complementary gains on top of PTID. On average, PTID improves the baseline by +13.8 mAP, +10.1 Rank-1, and +5.7 Rank-5. After further applying DVFC, the complete framework achieves average gains of +18.8 mAP, +11.5 Rank-1, and +6.9 Rank-5 over the original models.

The sequence-level results in Table 4 further show that the improvements are more pronounced when the baseline performance is relatively low. For example, the largest improvement is observed on RotTrans for Imsil-0824, where mAP increases from 59.4% to 94.3%, yielding a +34.9-point gain. The same setting also achieves the largest Rank-1 improvement, increasing from 68.2% to 94.2%, with a +26.0-point gain. This suggests that the proposed modules are particularly effective in improving initially weaker retrieval results.

Several observations can be drawn from these results. PTID contributes the majority of the total improvement, particularly in Rank-1, suggesting that pose- and text-conditioned generation enhances the identity representation. DVFC further improves performance, especially in mAP, indicating its effectiveness in refining the overall gallery ranking through viewpoint-aware neighborhood aggregation. Together, PTID and DVFC play complementary roles: the former strengthens identity representation through pose-guided generation, while the latter refines the retrieval feature distribution using reliable neighborhood structures. Their combination leads to consistent and substantial gains across closed-set sequences.

#### 3.3.2. Open-Set Evaluation

Under the open-set setting, we evaluate the proposed modules on four subsets with disjoint training and testing identities, as marked with an asterisk (*) in Table 5.

As shown in Table 5 and Table 6, the proposed modules consistently improve open-set identification performance across four ReID models. On average, PTID improves the baseline by +16.3 mAP, +8.4 Rank-1, and +3.7 Rank-5. After further applying DVFC, the complete framework achieves average gains of +24.9 mAP, +10.9 Rank-1, and +5.7 Rank-5 over the original models. The larger gap between PTID and PTID+DVFC in mAP suggests that viewpoint-aware neighborhood refinement is particularly useful for improving the overall gallery ranking when the testing identities are unseen during training.

The sequence-level results reveal a clear difference between the Imsil open-set sequences and the cross-region Namwon-0722* sequence. On Namwon-0722*, all models obtain large mAP gains after applying the proposed modules; for example, PFD improves from 54.4% to 95.2%, TransReID from 55.3% to 86.6%, and RotTrans from 55.9% to 95.7%. This suggests that the proposed framework is particularly effective when the test identities are unseen during training and collected from a different region. In contrast, the Imsil open-set sequences show lower initial mAP values and more moderate Rank-1 improvements, indicating that they remain challenging due to identity-level ambiguity among unseen cattle. The largest gains are observed on PFD, with a +40.8-point mAP improvement on Namwon-0722* and a +26.1-point Rank-1 improvement on Imsil-0823*. These results suggest that PTID enhances unseen-identity representation through pose-diverse support, while DVFC further stabilizes retrieval when the gallery contains visually similar cattle.

#### 3.3.3. Feature-Space Compactness and Separability Analysis

To quantitatively assess the learned identity representations, we report the mean intra-identity and inter-identity Euclidean distances, together with their ratio. A lower intra-identity distance indicates tighter clustering of samples from the same animal, while a lower intra/inter ratio reflects better relative compactness and separability. We also analyze positive- and negative-pair cosine similarity distributions and compute 95% confidence intervals for mAP and Rank-1 using identity-level bootstrap resampling with 1000 iterations.

**Figure 6:**
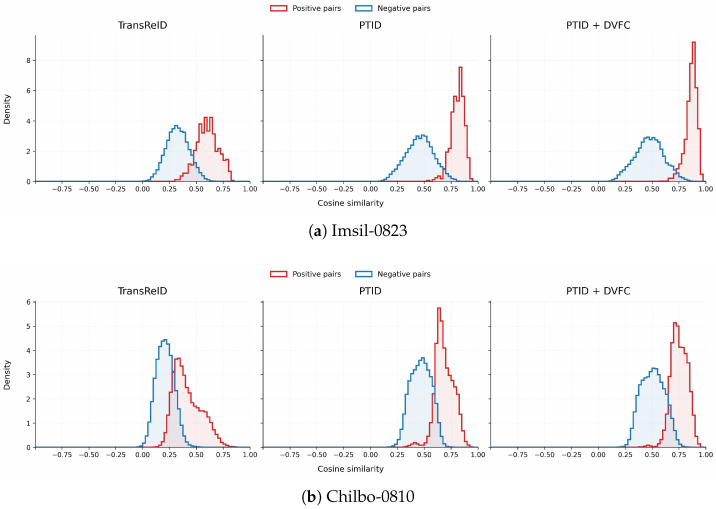
Positive- and negative-pair cosine similarity distributions obtained using TransReID, PTID, and PTID+DVFC on the Imsil-0823 and Chilbo-0810 sequences.

As shown in Table 7 and Figure 6, PTID consistently improves feature compactness on both sequences. On Imsil-0823, the intra-identity distance decreases from 0.88 to 0.59 after applying PTID and further to 0.43 with DVFC, while the intra/inter ratio decreases from 0.76 to 0.58 and 0.44, respectively. A similar trend is observed on Chilbo-0810. These results indicate that pose-diverse feature aggregation primarily reduces within-identity variation. Although the inter-identity distance also decreases, the larger reduction in intra-identity distance leads to better relative separability. After further applying DVFC, the lower intra/inter ratios suggest that viewpoint-aware neighborhood refinement further stabilizes the aggregated identity representations. Consistently, the positive-pair similarity distributions shift toward higher values and show less overlap with the negative-pair distributions, indicating clearer separation between same-identity and different-identity pairs. The identity-level bootstrap results provide additional support, with mAP increasing from 0.65 to 0.80 on Imsil-0823 and from 0.65 to 0.84 on Chilbo-0810, indicating that the improvements remain stable under identity-level resampling.

Overall, these results confirm that PTID improves intra-identity compactness, while DVFC further enhances the consistency and relative separability of the aggregated identity representations.

### 3.4. Qualitative Results

#### 3.4.1. Visualization of Generation Quality

**Figure 7:**
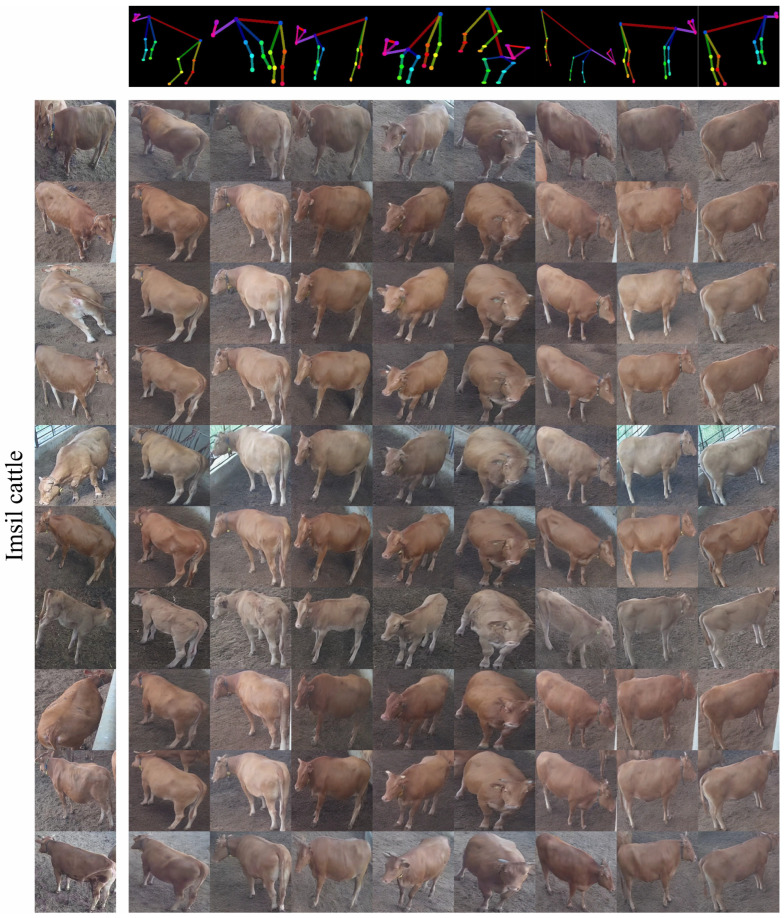
Image generation results in the Imsil scene. Benefiting from clearer reference images, the generated results preserve consistent appearance details and closely align with their visual characteristics.

**Figure 8:**
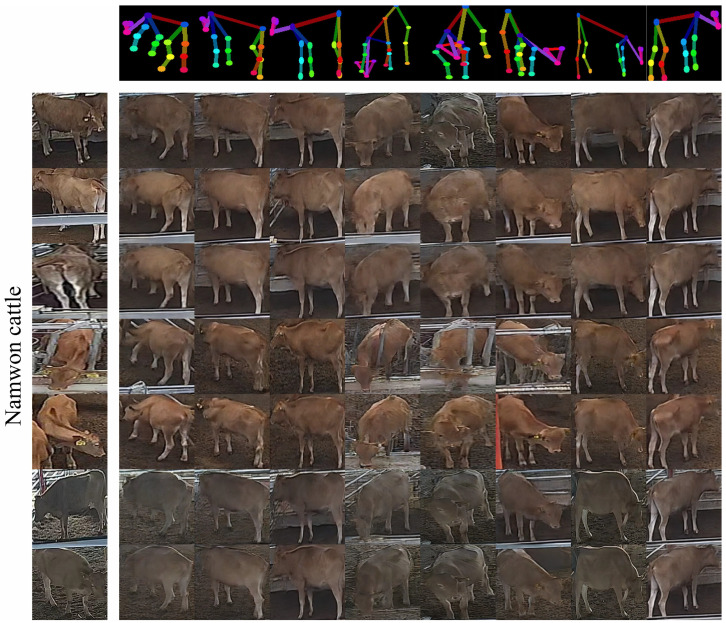
Image generation results in the Namwon scene. Due to lower camera resolution and longer capture distances, the visual quality of the generated images is limited. Nevertheless, fusing features from the reference and generated images remains effective in improving ReID performance under these challenging conditions.

**Figure 9:**
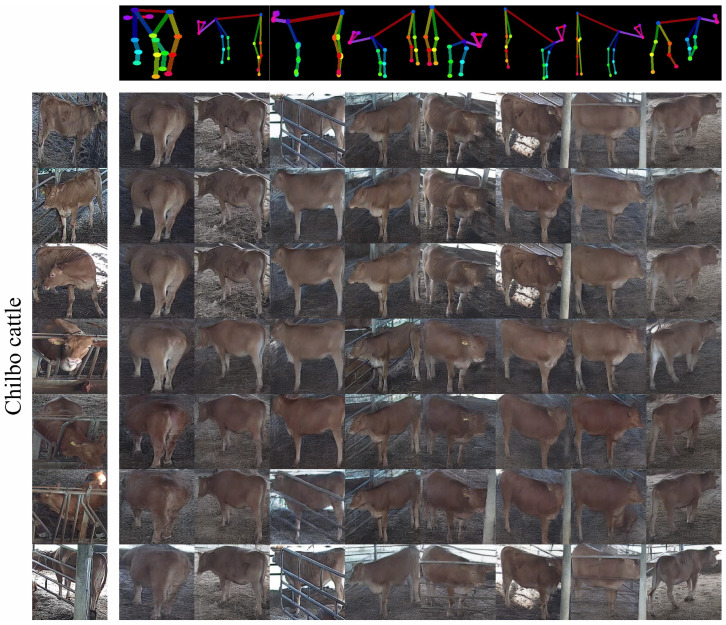
Image generation results in the Chilbo scene. Owing to occlusions, limited illumination, and missing key visual regions in the reference images, the perceptual quality of the generated results is constrained. Nevertheless, the generated samples still provide complementary cues that are beneficial for ReID at the feature level.

Figure 7, Figure 8 and Figure 9 present qualitative examples of Hanwoo image generation under eight representative poses and corresponding text prompts across three different scenes. Each row corresponds to one identity, where the leftmost column shows the reference image, and the remaining columns present generated images.

The Imsil scene provides relatively high-quality reference images, as the camera is installed above the cattle pens, resulting in clearer observations with fewer occlusions. Consequently, the generated images exhibit consistent appearance details, such as coat color, horn shape, and collars, closely matching the reference images. This indicates that the model effectively leverages both visual cues from the reference image and semantic information from the text prompts.

In contrast, the Namwon and Chilbo scenes are captured using a single distant camera, leading to limited viewpoint diversity. As a result, cattle are predominantly observed from fixed orientations, with side-view poses underrepresented. Generated images for these rare viewpoints tend to exhibit increased blur. Additionally, occlusions caused by fences, feed stacks, and body postures often obscure facial regions in the reference images. In challenging cases (e.g., when the cattle faces away from the camera), facial details in the generated results remain indistinct.

Overall, the Imsil scene achieves higher visual quality due to clearer and less occluded reference images. Although perceptual quality is degraded in the Namwon and Chilbo scenes due to occlusions and missing visual cues, incorporating the generated images consistently improves ReID performance, indicating that the synthesized samples provide complementary and discriminative information for inference-time feature aggregation.

#### 3.4.2. Visualization of Top-5 Retrieval Results

**Figure 10:**
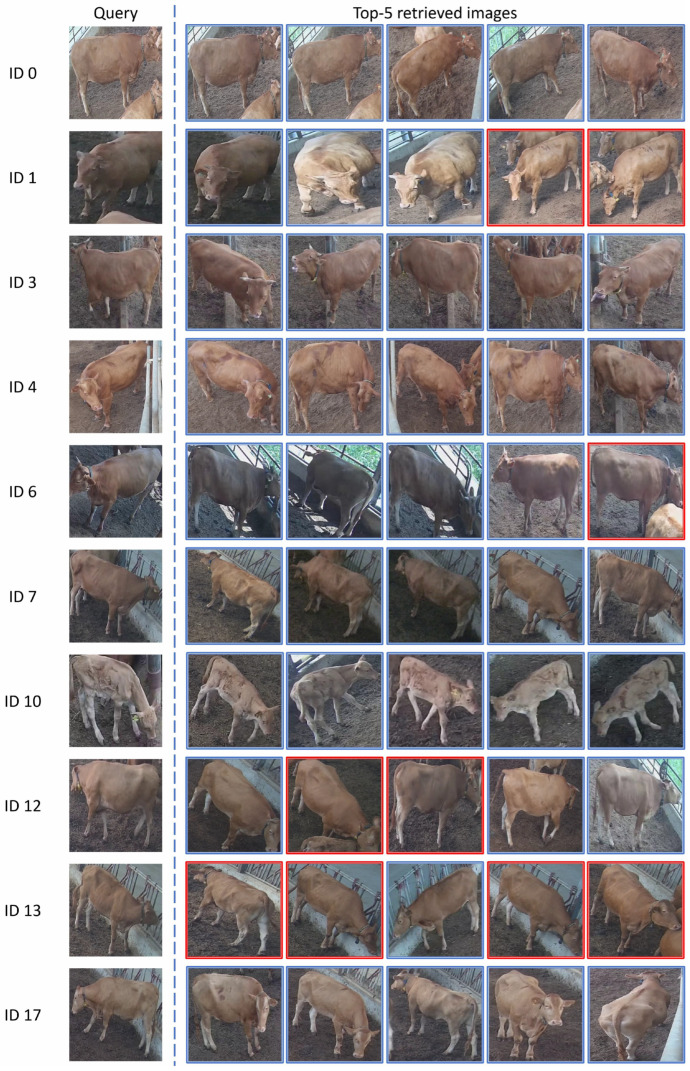
Visualization of top-5 retrieval results for representative query samples. Each row shows one query image and its top-5 retrieved gallery images. Blue borders indicate correct matches, while red borders indicate incorrect matches. The examples illustrate retrieval performance under substantial variations in viewpoint, pose, and appearance, including both successful and challenging cases in cross-view cattle ReID.

To provide an intuitive understanding of the retrieval behavior of the proposed method, we visualize the top-5 retrieved images for several representative query samples in Figure 10. Correct and incorrect matches are marked with blue and red borders, respectively. Most retrieved samples correspond to the correct identity despite substantial variations in viewpoint, body orientation, pose, and appearance, while several failure cases reveal the remaining ambiguity among visually similar cattle.

For example, in the ID 1 row, the fourth and fifth retrieved cattle differ from the query in horn appearance, while the fifth result for ID 6 also shows an inconsistent horn shape. Several incorrect results for ID 13 exhibit collar characteristics that differ from those of the query. In contrast, the ID 17 query contains more distinctive cues, including a relatively light coat, visible whitish facial regions, and ear tags, which facilitate more reliable matching. These examples show that cattle ReID cannot depend on a single visual attribute, since horn shape, collars, coat appearance, local markings, and pose provide complementary identity cues. This motivates the proposed framework to exploit visual, textual, and geometric information jointly. In particular, DVFC estimates body orientation and gives greater emphasis to features from viewpoint-consistent samples, since visual comparisons between cattle observed from similar orientations are generally more reliable than those across substantially different viewpoints. This viewpoint-aware refinement improves feature matching under cross-view and cross-pose variations.

#### 3.4.3. t-SNE Visualization of Feature Distributions

**Figure 11:**
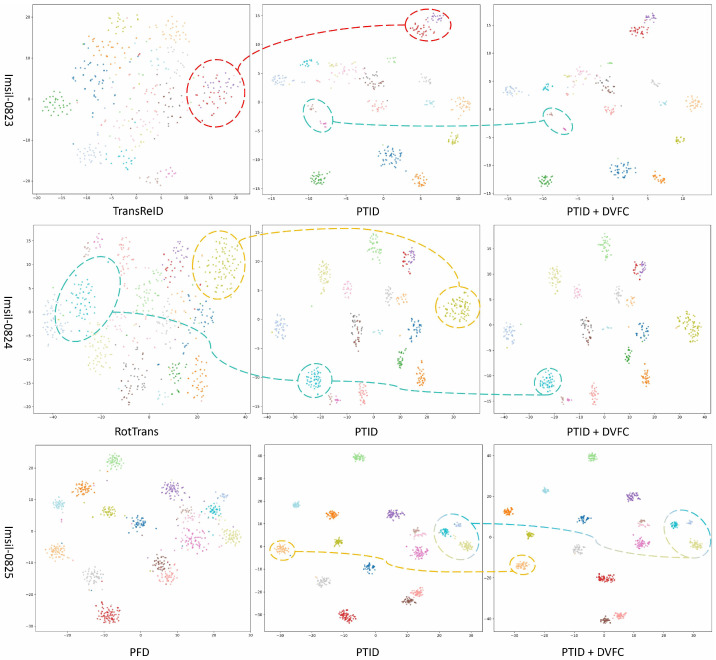
t-SNE visualization of feature distributions on Imsil-0823, Imsil-0824, and Imsil-0825. Each point represents one sample, and different colors denote different identities. From left to right, each row shows the baseline backbone, PTID, and PTID+DVFC. The highlighted regions illustrate the progressive transition from dispersed or partially mixed clusters to more compact and better-separated identity distributions.

**Figure 12:**
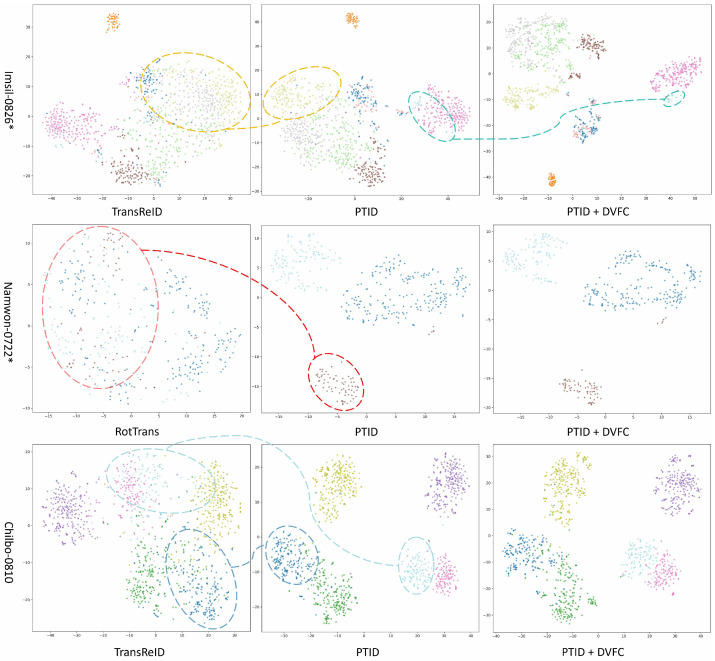
t-SNE visualization on more challenging sequences, including Imsil-0826*, Namwon-0722*, and Chilbo-0810. The dashed ellipses and connectors emphasize representative failure patterns of the baselines, such as severe overlap, elongated clusters, or ambiguous boundaries. PTID alleviates these issues by improving intra-class compactness, and DVFC further refines the local neighborhood structure to obtain cleaner cluster separation.

To qualitatively analyze the effect of each module, we visualize the feature distributions of selected sequences using t-SNE in Figure 11 and Figure 12. Although different rows correspond to different baseline backbones, a consistent trend can be clearly observed across all cases. Compared with the baseline features, PTID produces substantially more compact identity-wise clusters and alleviates the severe overlap between neighboring identities. In particular, several ambiguous regions highlighted by the dashed ellipses and connectors, which are highly scattered or mixed in the baseline feature space, become more organized after introducing PTID. This suggests that pose- and text-guided diffusion generation enriches cross-pose observations while preserving identity-consistent cues, thereby producing more compact identity representations through feature aggregation.

Based on PTID, DVFC further sharpens the cluster boundaries and reduces the residual interference between nearby identities. As shown in the highlighted regions, clusters that remain partially elongated, adjacent, or weakly mixed after PTID become cleaner and more isolated with PTID+DVFC. This effect is particularly evident on challenging cases such as Imsil-0823, Imsil-0824, Imsil-0826*, and Namwon-0722*, where the feature distributions evolve from heavily entangled patterns to much clearer identity-aware structures. Meanwhile, on Imsil-0825 and Chilbo-0810, where PTID already yields relatively well-separated clusters, DVFC still improves local margins and suppresses residual ambiguity. These observations demonstrate the complementary roles of the two modules: PTID enhances the global compactness of identity representations, while DVFC further improves local separability by refining the neighborhood structure in a viewpoint-aware manner.

### 3.5. Ablation Study

#### 3.5.1. Effect of Textual Conditioning

To evaluate the impact of textual conditioning in the proposed PTID model, we conduct an ablation study by comparing text-conditioned generation with a text-free counterpart under identical pose inputs. The generated images are evaluated using TransReID under the closed-set protocols on the Imsil, Namwon, and Chilbo datasets.

**Figure 13:**
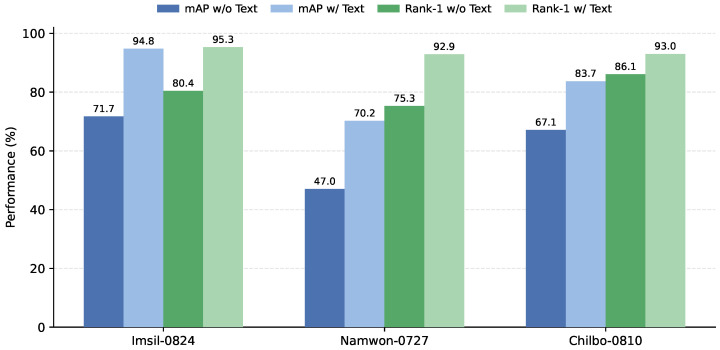
Ablation results of the proposed PTID model under text-conditioned and text-free settings, evaluated using mAP and Rank-1 on three datasets.

As shown in Figure 13, the grouped bars report the absolute mAP and Rank-1 values under the text-conditioned and text-free settings. Textual conditioning consistently improves both metrics across all datasets. On Imsil, it improves mAP and Rank-1 by 23.1 and 14.9 percentage points, respectively. Similar improvements are observed on Namwon, with gains of 23.2 and 17.6 points, and on Chilbo, with gains of 16.6 and 6.9 points, demonstrating consistent benefits across different scenes.

These results indicate that textual cues provide complementary semantic information beyond pose constraints, enabling the diffusion model to generate more identity-discriminative visual details. In contrast, without textual guidance, the generated images contain fewer distinctive appearance attributes, limiting their effectiveness for ReID despite accurate pose alignment.

#### 3.5.2. Effect on Cross-Scene Generalization

To evaluate the cross-scene generalization capability of PTID, we conduct cross-scene ReID experiments in which models trained on one scene are directly evaluated on unseen target scenes. Specifically, a model trained on Imsil is tested on Namwon-0727 and Chilbo-0810, while a model trained on Chilbo is evaluated on Imsil-0823. All experiments use the same TransReID backbone to ensure a fair comparison.

**Figure 14:**
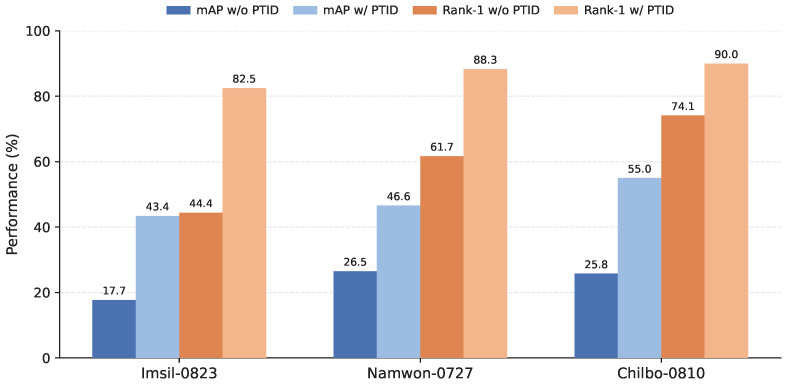
Cross-scene ReID performance with and without PTID on three target scenes, evaluated using a shared TransReID backbone in terms of mAP and Rank-1.

As shown in Figure 14, the grouped bars compare the absolute mAP and Rank-1 performance with and without PTID. PTID consistently improves both metrics across all cross-scene settings, indicating greater robustness under substantial domain shifts. The improvements are particularly pronounced in mAP, suggesting that PTID enhances overall ranking quality and retrieval consistency in unseen scenes.

These results suggest that PTID supports more transferable identity representations and improves ReID performance in previously unseen environments.

#### 3.5.3. Effect of Local and Global Neighborhood Sizes $k_{1}$ and $k_{2}$

We further analyze the influence of the neighborhood sizes used for local and global support in DVFC. As shown in Table 8, different $\left(k_{1},k_{2}\right)$ settings lead to different trade-offs across datasets and metrics. On Imsil-0823, $\left(k_{1},k_{2}\right)=\left(3,3\right)$ achieves the highest mAP of 88.9% and the strongest Rank-1 accuracy of 86.8%. Although enlarging the neighborhood to (3,5) or (5,5) slightly improves Rank-5 from 94.3% to 95.2%, it also leads to clear drops in mAP and Rank-1, suggesting that incorporating more neighbors may introduce less reliable contextual information. On Namwon-0727, (3,3) gives the best mAP of 81.0%, while (3,5) and (5,5) further improve Rank-1 from 93.5% to 94.8% with the same Rank-5 of 98.1%. These results indicate that increasing the number of neighbors may benefit top-ranked retrieval in some cases, but the effect is not consistent across datasets and metrics. Overall, these results further support the use of $\left(k_{1},k_{2}\right)=\left(3,3\right)$ as a balanced configuration that provides stable performance across datasets and evaluation metrics.

#### 3.5.4. Effect of Adaptive Support Weights $\alpha_{i}$ and $\beta_{i}$

We further evaluate whether sample-specific adaptive weighting is necessary for DVFC by comparing the proposed adaptive weights with a fixed-weight variant. As shown in Table 9, adaptive weighting consistently improves performance over fixed weighting across all evaluated sequences. On Imsil-0823, adaptive weights improve mAP from 88.4% to 88.9% and Rank-1 from 84.9% to 86.8%. On Imsil-0825, they bring larger gains of 1.8% in mAP, 4.4% in Rank-1, and 1.5% in Rank-5. On Namwon-0727, adaptive weighting yields a notable mAP gain of 3.9% while maintaining the same Rank-1 and Rank-5. On Chilbo-0810, it further improves mAP by 1.6% and Rank-5 by 1.0%. These results show that using shared fixed weights is insufficient to handle the varying reliability of local neighborhoods across samples. In contrast, the proposed adaptive weighting strategy can adjust the contributions of local and global support according to neighborhood reliability, leading to more robust and effective feature refinement.

#### 3.5.5. Effect of Local and Global Support Branches

We further analyze whether the local and global support branches are complementary by comparing the dual-branch design with its single-branch variants. Instead of reporting absolute performance, Table 10 summarizes the relative gains of the dual-branch DVFC over the local-only and global-only variants. Overall, combining the two support branches consistently improves performance across most datasets and metrics.

Compared with the local-only variant, the dual-branch design yields clear gains on all evaluated sequences, especially on the open-set sequences Imsil-0824* and Imsil-0826*, where it improves mAP by 1.6% and 0.1%, Rank-1 by 1.6% and 0.2%, and Rank-5 by 5.5% and 1.7%, respectively. Compared with the global-only variant, the dual-branch design still brings consistent Rank-1 improvements across all sequences, with gains of 2.0%, 1.0%, 1.6%, and 0.6%, respectively. These results indicate that the local branch provides finer viewpoint-aware cues, while the global branch offers more stable contextual support. Their combination, therefore, yields a more effective refinement strategy than either branch alone.

## 4. Discussion

This study improves visual ReID of weakly textured livestock by addressing the limited identity cues and substantial pose and viewpoint variations of Hanwoo cattle. Previous livestock identification methods have often relied on local biometric characteristics, including facial patterns [17,18,19], muzzle texture [20,21,22,23], and nose-print or related biometric cues [24,25]. Although effective under close-range and high-resolution imaging conditions, these cues may become unreliable in practical farm environments because of distance, occlusion, motion blur, and illumination variation. Global body-based ReID is, therefore, more compatible with camera-based livestock monitoring [3,26,27,28,29]. However, Hanwoo cattle present a particularly difficult case because their similar body appearance and weak texture provide only sparse identity-discriminative information.

Cross-view appearance variation further increases intra-identity feature dispersion and ambiguity among visually similar cattle. Earlier CNN-based studies demonstrated the feasibility of learning discriminative representations for animal identification [26,30,31], while Transformer-based methods improve robustness by modeling global relationships among image patches [11,12,32]. Camera/view information, patch-level transformations, and pose- or part-aware representations have also been shown to reduce feature ambiguity under viewpoint changes and occlusion [11,12,13,33]. Motivated by these observations, DVFC uses cattle orientation estimated from hoof geometry and evaluates neighborhood reliability before adaptively combining viewpoint-aware local support with global contextual support. This design is intended to reduce unreliable feature mixing across inconsistent viewpoints while preserving stable neighborhood information.

The results further indicate that generation-assisted feature aggregation is useful for ReID of weakly textured animals. Earlier generative models, including GANs and VAEs [34,35], established the potential of synthetic image generation, while diffusion models provide a more stable and controllable framework for image synthesis [14,36,37]. Latent diffusion and conditional generation methods further improve efficiency and controllability through compact latent representations and spatial guidance [14,38,39]. In this study, PTID combines a reference image, structural text guidance, and representative cattle poses to generate pose-diverse samples, whose features are aggregated with the original observation at inference time. Such generated samples are useful only when identity-discriminative cues are preserved, since effective ReID representations should reduce intra-identity variation while maintaining inter-identity separability [40]. Pose-guided generation has similarly been shown to improve identity representation by increasing pose diversity [7]. This is particularly important for Hanwoo cattle, whose subtle cues could be weakened by viewpoint changes or generation artifacts. PTID should, therefore, be understood as a representation-level enhancement strategy rather than a replacement for real observations.

The main computational overhead of the proposed framework comes from diffusion-based image generation. On a single NVIDIA H100 GPU, generating eight pose-diverse images for one input takes approximately one second, while the additional cost of DVFC is negligible. During PTID training with 256×256 images and a batch size of 64, the peak GPU memory usage is approximately 80 GB, which can be reduced by using a smaller batch size, gradient accumulation, or memory-saving techniques such as attention slicing and CPU offloading. Although the current implementation is not intended for continuous real-time inference on resource-constrained devices, it can be integrated into video-tracking systems, where ReID is mainly activated for identity reassociation after temporary tracking loss. Nevertheless, reducing generation latency remains an important direction for future work.

Several limitations remain. First, the current datasets contain a limited number of cattle identities, which restricts the evaluation of broader generalization. Second, the framework processes single images and does not fully exploit temporal information. Third, diffusion-based generation introduces substantial computational cost, limiting continuous real-time deployment on resource-constrained devices. Fourth, the structural text prompts require manual annotation, reducing scalability to open-world settings. Future work will focus on larger datasets, temporal and multi-camera integration, faster diffusion inference, and automatic prompt generation using vision-language models.

## 5. Conclusions

This work addresses ReID for Hanwoo cattle, where identity discrimination is challenging due to high inter-instance similarity, limited visual cues, and substantial pose and viewpoint variations. We proposed two complementary modules for improving cattle ReID. PTID generates pose-diverse images and aggregates features from the reference and synthesized samples to obtain a more compact identity representation, while DVFC performs lightweight viewpoint-aware feature refinement.

Experiments conducted on Hanwoo cattle from the Imsil, Namwon, and Chilbo farm environments showed that the proposed modules consistently improve multiple ReID backbones across different datasets and evaluation settings. The improvements in mAP and rank-based metrics demonstrate the effectiveness of combining pose-guided feature aggregation with viewpoint-aware refinement for Hanwoo cattle ReID in the evaluated environments.

These results support the effectiveness of the proposed framework for camera-based identification of Hanwoo cattle under the conditions considered in this study. Generalization to other animal species, weakly textured objects, and operational livestock monitoring systems remains to be validated. Future work will evaluate the framework on larger and more diverse datasets and investigate its integration into video-based ReID and multi-camera tracking systems.

## Figures and Tables

**Table 1:** Subset statistics of the Hanwoo ReID dataset, including scenes, sequences, cameras, identities, images, image resolution, and monitored area.

Scene	Sequence	Cameras	Identities	Images	Image Resolution	Area ($m^{2}$)
Imsil	0823	2	19	895	3840 × 2160	432
Imsil	0824	2	19	1449	3840 × 2160	432
Imsil	0825	2	19	2014	3840 × 2160	432
Imsil	0826	2	19	2723	3840 × 2160	432
Namwon	0722	1	3	850	1920 × 1080	100
Namwon	0727	1	9	1989	1920 × 1080	100
Chilbo	0810	1	6	2560	1920 × 1080	100
Total	–	–	37	12,480	–	–

**Table 2:** Evaluation protocols and train/query/gallery splits under the closed-set and open-set settings.

Setting	Evaluation Subset	Train Source	Query/Gallery Source	Train IDs	Test IDs	Train	Query	Gallery
Closed-set	Imsil-0823	Imsil^†^	Imsil-0823	19	19	3654	63	376
	Imsil-0824	Imsil^†^	Imsil-0824	19	19	3654	107	598
	Imsil-0825	Imsil^†^	Imsil-0825	19	19	3654	153	816
	Imsil-0826	Imsil^†^	Imsil-0826	19	19	3654	212	1102
	Namwon-0727	Namwon-0727	Namwon-0727	9	9	1029	154	806
	Chilbo-0810	Chilbo-0810	Chilbo-0810	6	6	1328	201	1031
Open-set	Imsil-0823*	Imsil^‡^	Imsil-0823*	10	9	3302	37	197
	Imsil-0824*	Imsil^‡^	Imsil-0824*	10	9	3302	127	565
	Imsil-0826*	Imsil^‡^	Imsil-0826*	10	9	3302	228	983
	Namwon-0722*	Namwon-0727	Namwon-0722	9	3	1029	67	342

Note: Imsil^†^ and Imsil^‡^ denote the merged Imsil training sets used in the closed-set and open-set protocols, respectively. Imsil^†^ uses all 19 Imsil identities, whereas Imsil^‡^ uses only the 10 open-set training identities. The symbol * denotes open-set evaluation with disjoint training and testing identities.

**Table 3:** Contribution summary under the closed-set setting. Results are averaged over the six sequences. $\Delta_{\mathrm{PTID}}$ denotes the gain brought by PTID over the baseline, and $\Delta_{\mathrm{PTID}+\mathrm{DVFC}}$ denotes the total gain of the complete framework over the baseline.

Backbone	mAP		Rank-1		Rank-5	
	$\Delta_{\mathrm{PTID}}$	$\Delta_{\mathrm{PTID}+\mathrm{DVFC}}$	$\Delta_{\mathrm{PTID}}$	$\Delta_{\mathrm{PTID}+\mathrm{DVFC}}$	$\Delta_{\mathrm{PTID}}$	$\Delta_{\mathrm{PTID}+\mathrm{DVFC}}$
TransReID	+15.3	+20.7	+10.7	+12.3	+6.7	+7.3
RotTrans	+16.7	+22.2	+12.5	+13.8	+6.1	+7.0
PFD	+13.7	+19.2	+10.4	+11.8	+5.4	+8.0
HanwooReID	+9.6	+13.0	+6.8	+8.2	+4.7	+5.4
Average	+13.8	+18.8	+10.1	+11.5	+5.7	+6.9

**Table 4:** Closed-set results on all sequences.

Backbone	PTID	DVFC	Imsil-0823			Imsil-0824			Imsil-0825			Imsil-0826			Namwon-0727			Chilbo-0810		
			mAP	Rank-1	Rank-5	mAP	Rank-1	Rank-5	mAP	Rank-1	Rank-5	mAP	Rank-1	Rank-5	mAP	Rank-1	Rank-5	mAP	Rank-1	Rank-5
TransReID	–	–	68.8	76.2	82.5	82.7	86.9	92.5	67.4	77.8	88.2	70.9	80.2	93.4	55.2	79.2	90.9	70.2	86.1	95.0
	✓	–	84.5	85.7	93.7	94.8	95.3	98.1	84.0	88.9	95.4	89.8	94.8	97.6	70.2	92.9	98.1	83.7	93.0	99.5
	✓	✓	88.9	86.8	94.3	97.4	98.1	99.0	90.9	92.3	97.2	92.9	95.1	98.0	81.0	93.5	98.1	88.4	94.5	99.5
RotTrans	–	–	68.7	77.8	85.7	59.4	68.2	88.8	72.1	80.4	92.2	80.8	89.2	98.1	70.9	77.9	87.0	47.8	72.6	89.6
	✓	–	81.9	81.0	93.7	88.2	91.6	97.2	90.2	92.8	98.7	95.3	97.6	99.1	79.1	89.0	92.2	65.2	89.1	97.0
	✓	✓	87.6	83.3	94.4	94.3	94.2	99.0	94.7	93.6	99.3	97.3	98.6	99.5	82.8	89.5	93.5	75.9	89.6	97.5
PFD	–	–	58.3	69.8	82.5	77.1	85.0	92.5	73.8	80.4	90.8	82.6	89.2	96.7	55.5	69.5	89.0	73.2	86.1	91.5
	✓	–	80.8	87.3	90.5	93.7	95.3	97.2	86.3	89.5	96.1	93.9	95.8	99.1	65.6	81.2	94.2	82.3	93.0	98.5
	✓	✓	89.8	88.5	100.0	97.7	97.1	99.0	92.5	90.7	96.4	96.5	96.1	100.0	73.6	85.1	96.1	85.9	93.0	99.5
HanwooReID	–	–	71.7	77.8	87.3	85.2	91.6	93.5	75.0	84.3	92.2	87.4	92.9	96.2	70.7	79.2	85.7	78.4	85.6	92.0
	✓	–	85.1	87.3	95.2	96.2	96.3	99.0	82.1	85.6	92.8	96.7	98.6	99.5	78.3	89.0	91.6	87.9	95.5	97.0
	✓	✓	90.7	88.5	96.2	98.0	97.1	99.0	88.2	91.7	93.1	98.5	99.0	100.0	81.2	89.0	93.5	89.9	95.5	97.5

**Table 5:** Open-set results on selected sequences.

Backbone	PTID	DVFC	Imsil-0823*			Imsil-0824*			Imsil-0826*			Namwon-0722*		
			mAP	Rank-1	Rank-5	mAP	Rank-1	Rank-5	mAP	Rank-1	Rank-5	mAP	Rank-1	Rank-5
TransReID	–	–	38.1	51.4	74.3	35.0	52.8	76.4	47.3	61.4	81.1	55.3	89.6	97.0
	✓	–	55.2	60.0	82.9	50.5	55.9	74.8	56.2	63.2	81.6	79.9	97.0	98.5
	✓	✓	67.1	64.3	85.7	54.7	59.5	78.6	62.2	63.2	82.5	86.6	97.0	100.0
RotTrans	–	–	36.8	40.0	77.1	31.1	48.0	75.6	45.2	56.1	80.7	55.9	86.6	95.5
	✓	–	50.7	57.1	85.7	44.5	52.2	81.1	51.3	62.7	82.9	89.4	98.5	100.0
	✓	✓	63.2	58.1	87.1	54.3	57.5	85.8	59.3	63.2	83.3	95.7	100.0	100.0
PFD	–	–	36.8	42.9	74.3	37.2	50.4	72.4	49.0	56.6	79.4	54.4	89.6	98.5
	✓	–	48.6	60.0	82.9	48.3	54.3	74.0	53.2	59.6	79.8	83.1	98.5	98.5
	✓	✓	63.3	69.0	86.2	56.1	58.3	81.1	60.1	59.8	81.1	95.2	100.0	100.0
HanwooReID	–	–	39.3	51.4	85.7	35.0	56.7	78.7	44.5	57.0	80.7	62.7	86.6	94.0
	✓	–	55.1	62.9	91.4	51.9	66.9	84.3	55.8	63.6	82.5	91.4	98.5	100.0
	✓	✓	66.7	71.4	92.9	60.2	66.9	85.0	60.3	65.4	82.9	96.8	98.5	100.0

**Table 6:** Contribution summary under the open-set setting. Results are averaged over the four open-set sequences. $\Delta_{\mathrm{PTID}}$ denotes the gain brought by PTID over the baseline, and $\Delta_{\mathrm{PTID}+\mathrm{DVFC}}$ denotes the total gain of the complete framework over the baseline.

Backbone	mAP		Rank-1		Rank-5	
	$\Delta_{\mathrm{PTID}}$	$\Delta_{\mathrm{PTID}+\mathrm{DVFC}}$	$\Delta_{\mathrm{PTID}}$	$\Delta_{\mathrm{PTID}+\mathrm{DVFC}}$	$\Delta_{\mathrm{PTID}}$	$\Delta_{\mathrm{PTID}+\mathrm{DVFC}}$
TransReID	+16.5	+23.7	+5.2	+7.2	+2.2	+4.5
RotTrans	+16.7	+25.9	+10.0	+12.0	+5.2	+6.8
PFD	+14.0	+24.3	+8.2	+11.9	+2.7	+6.0
HanwooReID	+18.2	+25.6	+10.1	+12.6	+4.8	+5.4
Average	+16.3	+24.9	+8.4	+10.9	+3.7	+5.7

**Table 7:** Quantitative comparison of feature-space compactness and separability, with identity-level bootstrap 95% confidence intervals. All configurations use TransReID as the backbone.

Sequence	PTID	DVFC	Intra Distance	Inter Distance	Intra/Inter	mAP (95% CI)	Rank-1 (95% CI)
Imsil-0823	–	–	0.88	1.15	0.76	0.65 [0.50, 0.80]	0.74 [0.56, 0.88]
	✓	–	0.59	1.03	0.58	0.78 [0.63, 0.90]	0.78 [0.61, 0.94]
	✓	✓	0.43	1.00	0.44	0.80 [0.67, 0.92]	0.78 [0.62, 0.92]
Chilbo-0810	–	–	1.08	1.25	0.86	0.65 [0.49, 0.83]	0.82 [0.67, 0.94]
	✓	–	0.79	1.03	0.77	0.81 [0.71, 0.91]	0.91 [0.84, 0.96]
	✓	✓	0.62	0.96	0.65	0.84 [0.78, 0.91]	0.95 [0.91, 0.98]

**Table 8:** Ablation study on the neighborhood sizes $k_{1}$ and $k_{2}$ in DVFC.

Sequence	$k_{1}$	$k_{2}$	mAP	Rank-1	Rank-5
Imsil-0823	1	1	87.9	82.0	94.3
	3	3	88.9	86.8	94.3
	3	5	87.3	83.9	95.2
	5	5	87.3	83.9	95.2
Namwon-0727	1	1	77.9	90.9	97.4
	3	3	81.0	93.5	98.1
	3	5	79.7	94.8	98.1
	5	5	79.7	94.8	98.1

**Table 9:** Ablation study on the adaptive support weights in DVFC. Fixed weights use shared α and β for all samples, while adaptive weights determine $\alpha_{i}$ and $\beta_{i}$ according to neighborhood reliability. Gains are computed as adaptive minus fixed.

Sequence	Fixed Weights			Adaptive Weights			Gain		
	mAP	Rank-1	Rank-5	mAP	Rank-1	Rank-5	ΔmAP	ΔRank-1	ΔRank-5
Imsil-0823	88.4	84.9	94.3	88.9	86.8	94.3	+0.5	+1.9	+0.0
Imsil-0825	89.1	87.9	95.7	90.9	92.3	97.2	+1.8	+4.4	+1.5
Namwon-0727	77.1	93.5	98.1	81.0	93.5	98.1	+3.9	+0.0	+0.0
Chilbo-0810	86.8	94.5	98.5	88.4	94.5	99.5	+1.6	+0.0	+1.0

**Table 10:** Relative gains of the dual-branch DVFC over the local-only and global-only variants. Positive values indicate that combining local and global support improves performance.

Sequence	Improvement over Local-Only			Improvement over Global-Only		
	ΔmAP	ΔRank-1	ΔRank-5	ΔmAP	ΔRank-1	ΔRank-5
Imsil-0823	+1.6	+0.7	+2.0	+0.4	+2.0	+0.0
Imsil-0824	+0.3	+1.0	+0.0	+0.0	+0.0	+0.0
Imsil-0824*	+1.6	+1.6	+5.5	+0.5	+1.6	+0.0
Imsil-0826*	+0.1	+0.2	+1.7	+0.1	+0.6	+0.4

## Data Availability

The dataset will be made available on request.
